# Slab gliding, a hidden factor that induces irreversibility and redox asymmetry of lithium-rich layered oxide cathodes

**DOI:** 10.1038/s41467-023-39838-y

**Published:** 2023-07-12

**Authors:** Jun-Hyuk Song, Seungju Yu, Byunghoon Kim, Donggun Eum, Jiung Cho, Ho-Young Jang, Sung-O Park, Jaekyun Yoo, Youngmin Ko, Kyeongsu Lee, Myeong Hwan Lee, Byungwook Kang, Kisuk Kang

**Affiliations:** 1 Department of Materials Science and Engineering, Research Institute of Advanced Materials (RIAM), Seoul National University, 1 Gwanak-ro, Gwanak-gu, Seoul 151-742 Republic of Korea; 2 Western Seoul Center, Korea Basic Science Institute, 150 Bugahyeon‐ro, Seoul, 03759 Republic of Korea; 3 Center for Nanoparticle Research, Institute for Basic Science (IBS), Seoul National University, 1 Gwanak-ro, Gwanak-gu, Seoul 151-742 Republic of Korea; 4 Institute of Engineering Research, College of Engineering, Seoul National University, 1 Gwanak-ro, Gwanak-gu, Seoul 151-742 Republic of Korea; 5 School of Chemical and Biological Engineering, and Institute of Chemical Process, Seoul National University, Seoul, 08826 Republic of Korea; 6 Present Address: LiB Materials Research Group, Research Institute of Industrial Science & Technology (RIST), 100 Songdogwahak-ro, Yeonsu-gu, Incheon Republic of Korea; 7 Present Address: Department of Advanced Materials Engineering, Chung-Ang University, 4726, Seodong-daero, Daedoek-myeon, Anseong-si, Gyeonggi-do 17546 Republic of Korea

**Keywords:** Batteries, Batteries

## Abstract

Lithium-rich layered oxides, despite their potential as high-energy-density cathode materials, are impeded by electrochemical performance deterioration upon anionic redox. Although this deterioration is believed to primarily result from structural disordering, our understanding of how it is triggered and/or occurs remains incomplete. Herein, we propose a theoretical picture that clarifies the irreversible transformation and redox asymmetry of lithium-rich layered oxides by introducing a series of global and local dynamic structural evolution processes involving slab gliding and transition-metal migration. We show that slab gliding plays a key role in trigger/initiating the structural disordering and consequent degradation of the anionic redox reaction. We further reveal that the ‘concerted disordering mechanism’ of slab gliding and transition-metal migration produces spontaneously irreversible/asymmetric lithiation and de-lithiation pathways, causing irreversible structural deterioration and the asymmetry of the anionic redox reaction. Our findings suggest slab gliding as a crucial, yet underexplored, method for achieving a reversible anionic redox reaction.

## Abstract (web summary)

Lithium-rich layered oxides, promising high-energy-density cathode materials, face electrochemical degradation due to structural disordering. Here, the authors report that slab gliding is a key trigger of this disordering and a pivotal unexplored avenue for a reversible anionic redox reaction.

## Introduction

For further expansion of the electric mobility market, it is indispensable to substantially enhance the energy density of conventional lithium-ion batteries, which requires innovations in the electrode chemistry^1,2^. Lithium-rich layered oxides are regarded as promising next-generation cathodes with exceptionally high-energy density^3^, as they can harness the capacity from the anionic oxygen-redox activity in addition to that from the cationic redox reaction^4^. However, the use of the oxygen-redox in lithium-rich layered oxides has been limited by intrinsic shortcomings with respect to stability, as these materials display a large voltage suppression/hysteresis and capacity loss as cycling progresses, which have been contributed to the gradual structural deterioration of the host material upon the oxygen redox^5–9^.

Transition metal (TM) displacement in the lithium-rich layered structure has been highlighted as a representative type of structural disordering discovered during the oxygen-redox reaction. Lithium-rich layered oxide electrodes have been reported to undergo a significant extent of out-of-plane TM migration at the first charge process^7,10^. In addition, these TM migrations restore partially reversible during the subsequent discharge, with this irreversibility steadily increasing over subsequent cycles^11^. It has also been observed that TM migration can arise in the lateral direction, with TM ions moving to the vacancies within the same TM slab^12,13^. Such in-plane TM migration was initially found in P-type Na-based oxygen-redox electrodes, where out-of-plane TM migration is generally prohibited by the discrepancy between TM ions and Na prismatic sites^12^. However, recent studies further revealed that these in-plane structural disorderings could not be recovered, gradually disordering the original layered structure as cycling progressed^14^. Similar phenomena were also observed in conventional O-type lithium-based electrodes^13^. More importantly regarding these irreversible TM migrations is that they are closely related to the incessant degradation of the electrochemical properties. It is because the TM displacement and corresponding structural disorderings cause the change in the redox capability of both oxygen and TM, thereby continuously affecting the deliverable capacity and voltage during repeated cycles^7,10,11^.

**Fig. 1:**
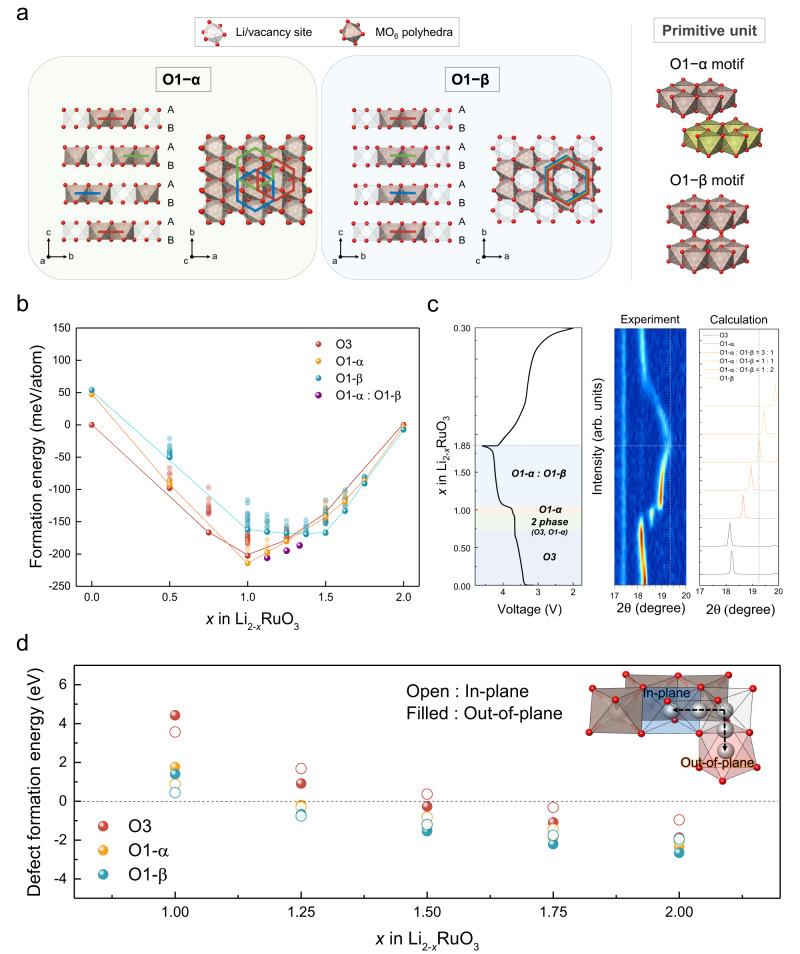
Trends of stacking sequence changes and defect formation in Li_2−*x*_RuO_3_ (0 ≤ *x* ≤ 2). **a** Different O1–α and O1–β stacking structures and motifs. **b** Formation energies of each stacking sequence (O3, O1–α, O1–β, hybrid O1–α and O1–β) depending on lithium content. According to the energies of the O3 stacking sequence, the relative energies are provided. For each lithium contents, the most stable energy for the various lithium configurations is displayed in bold, while the rest are dimmer. **c** (left) First cycle profile of Li_2−*x*_RuO_3_ electrode (0 ≤ *x* ≤ 1.85) in lithium cell. (middle) In situ XRD pattern of Li_2−*x*_RuO_3_ electrode (0 ≤ *x* ≤ 1.85) during the electrochemical cycle. (right) Calculated (003) XRD peaks for O3- and O1-Li_2−*x*_RuO_3_ (0 ≤ *x* ≤ 1.85). **d** Calculated TM migration energies for Li_2−*x*_RuO_3_ (1 ≤ *x* ≤ 2). Depending on lithium content, only the migration energies that are the most stable are displayed. In-plane migration means that the Ru ion migrates to the neighboring unoccupied octahedral position in the identical TM slab. Out-of-plane migration means that the Ru ion migrates to the neighboring unoccupied tetrahedral position in the lithium slab for the O3 phase and to the unoccupied octahedral position in the lithium slab for the O1–α and O1–β phases.

Previously, we proposed that the TM migration can be structurally regulated within the layered structure by altering the oxygen stacking sequences from O3- to O2-type in lithium-rich layered oxides^14^. Even though the out-of-plane TM migration may occur to some extent, it could be tailored in a reversible manner in the O2-type layered structure during the charge/discharge, which aided in inhibiting the irreversible degradation of the material, thereby preserving the voltage retention. This finding demonstrated the importance of controlling the local TM migrations in the lithium-rich layered material in stabilizing the oxygen-redox activity. Nevertheless, it also infers that the local TM migrations are indeed sensitively affected by the global structural framework such as stacking sequences in the layered structure. Since slab gliding occurs during the de-/lithiation process in the structures of most layered electrode materials, it implies that controlling of the reversibility of the local TM migrations may not be sufficient in securing the overall structural reversibility and oxygen activity. The slab gliding from O3-to O1 has been frequently observed for a variety of lithium-stoichiometric layered materials (e.g., LiCoO_2_ and LiNiO_2_)^15–17^. In addition, more diverse stacking variations are possible in lithium-rich layered oxides due to the presence of the vertical arrangement of honeycomb superstructures (e.g., O1–α and O1–β), as illustrated in Fig. 1a and S1^17–19^. The presence of this additional factor indicates an even higher TM migration complexity and its reversibility in the dynamic de-/lithiation process. Of great significance is that depending on the combinations of lateral slab gliding and out-of-plane/in-plane TM migration, various cation migration pathways may exist, complicating the possible restoration of the initial cation arrangements during oxygen-redox (i.e., structural reversibility). Despite these implications, it has not yet been elucidated how structural and electrochemical reversibility are related to the dynamic correlations between slab gliding and TM migration.

In this work, we demonstrate a structural model of the correlation among factors that induce local and global transitions during the oxygen-redox, which clarifies the complex phase transition of lithium-rich layered oxides. It is shown that slab gliding, a lateral structural shift that occurs during de-/lithiation, combines with local disorderings such as out-of-plane/in-plane TM migration to yield various reversible/irreversible transition pathways of the structural evolutions. We observe that a significant number of the irreversible/asymmetric pathways are spontaneous and gradually deteriorate the structural integrity of the layered materials, producing various disorder motifs that have been experimentally reported^6,7,9,10,13,20,21^. More importantly, the formation of these disordered structures is most favorably triggered via pathways of slab gliding-mediated TM disorderings than by the direct TM migration, indicating the importance of the concerted disordering mechanisms. Our findings not only suggest a model system for the irreversible and complex structural transition of lithium-rich layered oxides but also unveil that the slab gliding control in the layered structure may be an unexplored yet important route toward achieving a reversible oxygen-redox reaction.

## Results

### Simultaneous occurrence of slab gliding and TM migration

We selected Li_2−*x*_RuO_3_ as a model lithium-rich electrode system to investigate the global and local structural transitions, as it has been reported to exhibit substantial voltage suppression/hysteresis along with disintegration of the layered structure upon oxygen-redox^6,21,22^. First, we examined the potential alternations of the stacking sequence in the layered Li_2−*x*_RuO_3_ structure depending on lithium content ($0\le x\le 2$) by probing the relative formation energies, as illustrated in Fig. 1b. Various layered stackings that can be produced from the original O3 structure were considered, including O1–α, O1–β, and hybrid structures of the two stackings (see Fig. S2 for details). O3 stacking reveals the most stable structure in the cationic Ru^4+/5+^ redox region during the early charging stage ($0\le \, x \, < 1$)^21,23^. However, when further de-lithiation was performed, the O1–α stacking became more stable and eventually the most energetically favorable phase at $x\ge 1$, suggesting the O3-to-O1 structural transition, consistent with previous research^21^. Notably, we observed that upon further de-lithiation, the O1–α phase is transformed gradually to O1–β phases ($x \, > \, 1.5$), undergoing the stages of the hybrid structures of O1–α and O1–β in the range of $1 \, < \, x \, < \, 1.5$ (purple dots). This conversion from O1–α to O1–β phase has not been theoretically or experimentally observed to date. We attempted to experimentally verify this conversion by in situ X-ray diffraction (XRD), as depicted in Fig. 1c and S3 (experimental details can be found in the supplementary information). The figure demonstrates the maintenance of the O3 stacking until Li_1.25_RuO_3_ during charge process with a slight peak shift, which corresponds to the interlayer distance increasing. Afterwards, the O1–α phase begins to appear at a characteristic peak position of 2${{{{{\rm{\theta }}}}}}$ = 18.59°^21^, at the expense of the O3 phase. Upon further de-lithiation, we could confirm that the O1–α phase is converted into the O1–β phase, consistent with our theoretical prediction. A new peak appeared at 2${{{{{\rm{\theta }}}}}}$ = 18.94°, substantially differing from the characteristic O1–α peak at 18.59°, when charged to $x \, > \sim 1$, and the peak gradually shifted to higher angle at the end of the charge ($x \sim 1.85$). According to our XRD pattern simulations (right panel in Fig. 1c), the new peak (2${{{{{\rm{\theta }}}}}}$ = 18.94°) can be assigned to hybrid structures of the O1–α and O1–β phases, with the peak shift toward higher angle attributed to the increasing proportion of the O1–β phase (see supplementary information S4 for details).

It is noteworthy that Ru migration was previously observed during oxygen-redox in the region where the O1–α to O1–β transition has occurred ($1 \, \le \, x \, \le \, 2$)^6,21^. Our density functional theory (DFT) calculation also confirms that both the in-plane and out-of-plane migrations are energetically plausible in the de-lithiated Li_2−*x*_RuO_3_ phase with O1–α or O1–β stackings ($1 \, < \, x \, \le \, 2$), as evidenced by the defect formation energies in Fig. 1d. More importantly, this finding suggests that the likelihood of Ru migration can be significantly affected by the initial stacking sequences. Figure 1d illustrates that the spontaneous in-plane or out-of-plane migration occurs for O1–α and O1–β as early as $x=1.25$, whereas it is not energetically permitted for O3-Li_0.75_RuO_3_. Even in the highly de-lithiated state, where Ru migration appears to be favorable regardless of the stacking sequences, it is observed that O1–α and O1–β are more prone to in-plane or out-of-plane Ru migration than the O3 phase. Taken together, Ru migration can happen spontaneously in the oxygen-redox region of Li_2−*x*_RuO_3_ (1 < *x* ≤ 2), accompanied by the occurrence of O1–α and O1–β stacking shift, indicating the need to understand the dynamic interplay between the change of stacking and TM migration behavior.

### Asymmetric pathways and structural irreversibility

**Fig. 2:**
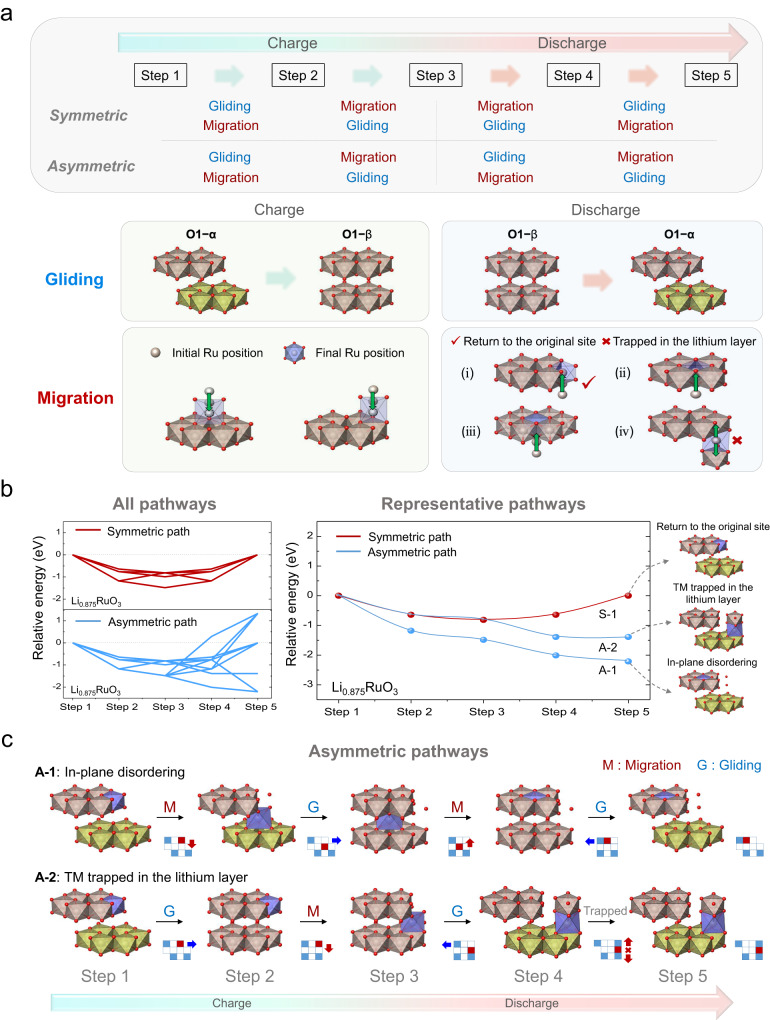
Various pathways caused by combinations of slab gliding and TM migration during charge/discharge. **a** Combination in sequence of slab gliding and TM migration during an electrochemical charge–discharge cycle. Depending on the sequencing of each gliding/migration, the combinations are split into two sets of four cases, symmetric and asymmetric sequences. During the charging process, slab gliding from O1−α to O1−β occurs, whereas out-of-plane TM migration to the lithium layer can result in two types of neighboring conditions (face shares with vacancy or transition metal). During the discharging process, slab gliding occurs from O1−β back to O1−α, while the TMs can re-migrate to occupy four different types of sites (i: return to the original site, ii: surrounded by 5 TM ions, iii: surrounded by 6 TM ions, iv: TM trapped in the lithium layer). **b** (left) The energy landscape of all possible pathways (symmetric and asymmetric). (right) The energy landscape of representative pathways (symmetric and asymmetric). The representative symmetric path is denoted as S-1, and the asymmetric paths are denoted as A-1 and A-2, respectively. The final structure motifs via symmetric and asymmetric pathways are depicted on the right. **c** Schematic illustration of representative asymmetric structural evolution through consecutive combination of gliding and out-of-plane TM migration (A-1, A-2). Each step was illustrated by a motif that represented the structural configuration. Between each step, a Lego-type picture is provided for a better illustration of the structural transformation, where the red, blue, and white squares refer migrating TMs, occupied TMs, and vacant sites, respectively. Additionally, red arrows show TM migration, whereas blue arrows imply slab gliding.

The simultaneous occurrence of slab gliding and out-of-plane migration can lead to a diversity of structural transformation pathways during the charge–discharge process. In this respect, we systematically investigated scenarios of de-/lithiation processes in the oxygen-redox region of O1-Li_2−*x*_RuO_3_ ($x \, > \sim \,1$), which involves sequential steps of (i) slab gliding between O1–α and O1–β and (ii) out-of-plane Ru migration, as shown in Fig. 2a. Steps 1→3 and 3→5 correspond to potential structural disorderings that can occur during charge and discharge of O1-Li_2−*x*_RuO_3_, respectively, involving the gliding and migration. In each process, the slab gliding and Ru migration were assumed to occur alternatively, which gives rise to two cases of either symmetric or asymmetric sequential charge/discharge process. Considering (i) the slab gliding between O1–α and O1–β phases and (ii) Ru migrations represented during a charge/discharge cycle, a total of 20 individual paths of intra-cycle symmetric (5) or asymmetric (15) structural transformations were identified for Li_2−*x*_RuO_3_ (all the detailed paths are schematically illustrated in Figs. S6–8 and tabulated in Table S1). In an ideal case, the combined migration and gliding can occur during charge and discharge in the exact opposite way consecutively (see the upper panel in Fig. 2a), retaining the original layered structure after a cycle, as indicated by the red check mark in Fig. 2a (i). However, certain combinations of slab gliding and TM migration sequences can result in an asymmetric process of charge and discharge. For example, the gliding from O1–α to O1–β occurs during charging, followed by Ru ion migration in the O1–β structure; however, the discharge process can be initiated by the reverse gliding process first, leaving the migrated Ru trapped in the lithium layer (marked with a red x symbol in Fig. 2a (iv)), as will be discussed in detail later.

In Fig. 2b (left), the energy landscapes of all the pathways (5 symmetric and 15 asymmetric pathways) are plotted with respect to disordering steps 1–5 in Fig. 2a. All the paths display a downhill energy profile during the charging process (steps 1–3), indicating the thermodynamic spontaneity of the combined out-of-plane Ru migration and slab gliding in O1-Li_2−*x*_RuO_3_. However, during the discharge process (steps 3–5), this spontaneity serves as an energy penalty in recovering the original layered structure in the symmetric paths. However, we observed that some asymmetric pathways exhibit downhill-reaction energetics even during the discharge process, indicating that these asymmetric pathways can trigger the intra-cycle irreversibility of the layered framework.

To understand the detailed mechanism of structural evolution, we selected two representative asymmetric pathways (denoted as A-1 and A-2) and one symmetric pathway (denoted as S-1), whose energy landscapes are re-plotted in the right panel of Fig. 2b. In the case of the symmetric path (S-1), the sequence and direction of slab gliding and TM migration are exactly opposite for charge and discharge processes (see Fig. S9 for details), indicating that the electrode may recover its original structure after an electrochemical cycle with an energy barrier of ~<1 eV. However, in the asymmetric paths, slab gliding and TM migration do not occur in the same sequential manner upon charge and discharge, resulting in layered structures that differ from the pristine structure. In the asymmetric pathway A-1 (Fig. 2c (top panel)), the Ru migration is followed by slab gliding during charging, whereas the discharge involves the Ru migration back to the TM layer before the gliding occurs, effectively inducing the ‘in-plane disordering’^12,13^ within the TM layer at the lower energy state than that of pristine structure. In another downhill asymmetric path of A-2, Ru can occupy the lithium layer after slab gliding during a charge process, as depicted in Fig. 2c (bottom panel); however, slab gliding may occur initially during discharge such that the migrated Ru is blocked by both the upper and lower TM layers. In this situation, the return of the Ru ion to the original TM layer becomes inaccessible, consequently resulting in the ‘TM trapped in the lithium layer’^6^ state after discharging. These asymmetric pathways are energetically spontaneous processes throughout all the individual steps, which may account for the experimentally observed ‘in-plane disordering’^12,13^ and ‘TM trapped in the lithium layer’^6^ in lithium-rich materials after cycling.

### Redox asymmetry arising from asymmetric structural transformations

**Fig. 3:**
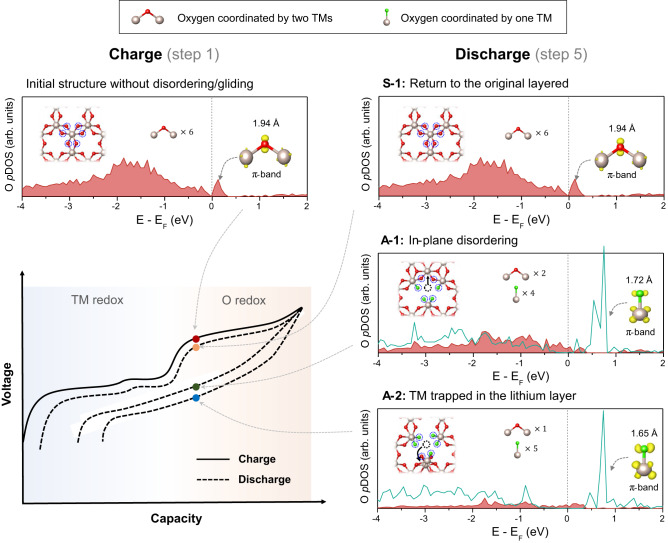
Electronic structure comparison of the initial (step 1) and final (step 5) configurations created by successive repetition of slab gliding and TM migration. Blue dotted circles in the each graph inset denote six oxygens of interest, including a red atom coordinated to two TMs and a green atom coordinated to one TM. The total of the oxygen electron densities with the same coordination is represented by the same hue as each oxygen. Additionally, the shortest TM–O bond length of each local structure and corresponding electron densities are represented in the right side inset. The voltage profile, positioned on the lower left side, illustrates schematic diagrams for each case (S-1, A-1, and A-2).

We speculated that this spontaneous evolution of the Li_2−*x*_RuO_3_ structure would affect the oxygen-redox activity upon cycling as well as the electrochemical reversibility. In this regard, the extent of its effect was investigated by comparatively revisiting the electronic structure around the oxygen considering the representative symmetric and asymmetric structural transformations. Figure 3 displays the oxygen projected density of states (O pDOS) calculated for the oxygen-redox region during charge (solid line of electrochemical profile in the lower left figure) and discharge with each disordering scenario (dotted lines). The O pDOS diagram on the right depicts the electronic structure of the final stage of the structural evolution for the S-1, A-1, and A-2 transformations, respectively, whereas that on the left shows the original O pDOS state before the disordering/gliding (see Methods for details). For clarity, the oxygen coordinated by two Ru ions or one Ru ion is represented by red and green spheres, respectively, in the inset of pDOS, and the corresponding electron density contribution from each oxygen atom is displayed in red (filled area) and green (unfilled area), respectively. In the pristine structure, all the oxygen bond to two Ru ions with a bond length of approximately 1.94 Å in Li_0.875_RuO_3_. The pDOS clearly demonstrates that compensation of charge occurs in the oxygen-property-dominant π-band above the Fermi energy level (dotted line), which is consistent with a previous study^24–26^. When the symmetric structural transformation (S-1) is completed after an electrochemical cycle, it recovers the initial electronic structure of the pristine state, implying that the charge and discharge profile will be symmetric in shape with only a small hysteresis, as indicated by the red and orange dots in the figure. However, if the final structure evolves along an asymmetric path (A-1 or A-2) to ‘in-plane disordering’ or ‘TM trapped in the lithium layer’, it was observed that single-coordinated oxygens were prone to be generated (green atom in the inset). Furthermore, the single-coordinated oxygen formation shortens the Ru–O distance, 1.72 Å for ‘in-plane disordering’ and 1.65 Å for ‘TM trapped in the lithium layer’, which are substantially shorter than the distance of 1.94 Å in the pristine structure. This difference is observed because the oxygen atom that loses one of its two bonds with Ru forms a stronger interaction with the bonded Ru coordination.

The π-type metal–oxygen bonding strength generally increases as the atomic orbital overlay increases. It suggests that a shorter Ru–O bond distance would induce greater π-type interaction, thereby shifting the antibonding level to a higher energy, leading to an electronic structural rearrangement^27^. Consistent with the bond lengths difference, the ‘in-plane disordering’ structure with the shortened Ru–O distance of 1.72 Å exhibits a higher energy state than the antibonding level of the pristine structure in the pDOS (Fig. 3A-1). Similarly, the antibonding level of the ‘TM trapped in the lithium layer’ with the shortest Ru–O distance of 1.65 Å was moved to the highest energy state among the three structures (Fig. 3A-2), which translates into the largest drop in the overall potential of discharge, as depicted by the blue dots in the electrochemical profile. This finding clearly manifests that the asymmetric transformation of the layered structure induces the significant alternation in the oxygen bonding nature (e.g., bond length/strength with Ru and the emergence of single-coordinated oxygens), which leads to substantial suppression of the discharge voltage and the corresponding voltage hysteresis in the cycle.

### Slab gliding links in-plane and out-of-plane disorderings

**Fig. 4:**
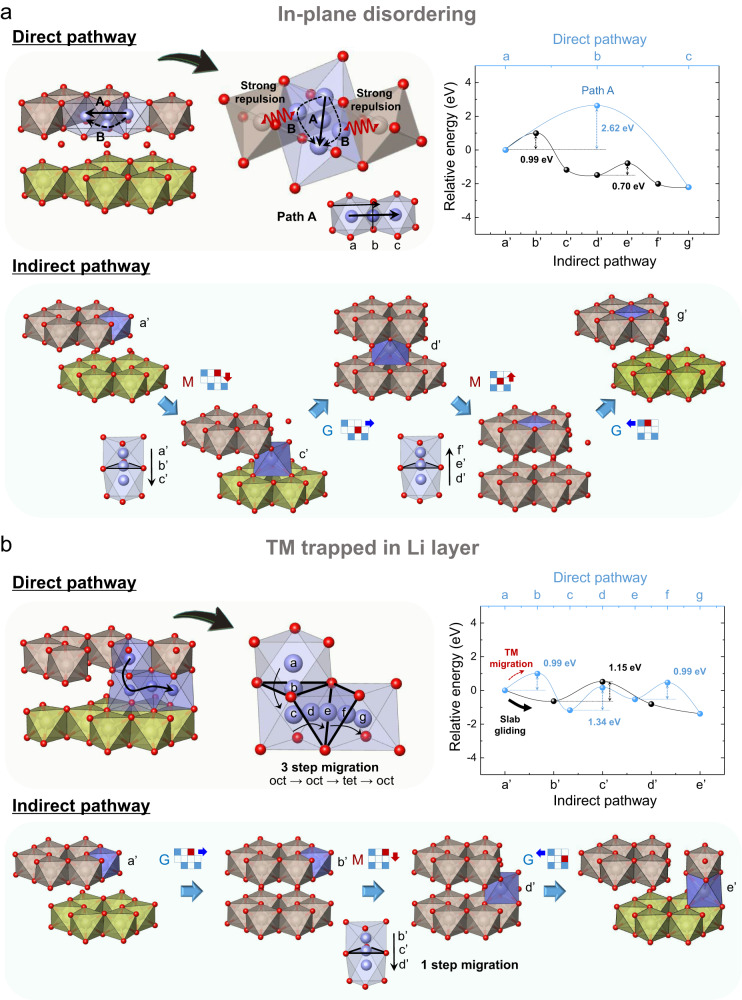
Energetics comparison of direct and indirect structural transformation paths for ‘in-plane disordering’ and ‘TM trapped in the lithium layer’ structure. **a** Two distinct scenarios occuring the ‘in-plane disordering’ structure. (Top panel) Direct pathway employing TM ion in-plane hopping. (Bottom panel) Indirect pathway employing slab gliding and hopping in the direction of out-of-plane. The moving cation’s positions are indicated by points a–c and a’–g’. (Right) The calculated energy landscape of two pathways, direct and indirect, depicted in the panels. **b** Two distinct scenarios occuring the ‘TM trapped in the lithium layer’ structure. (Top panel) Direct pathway employing the tetrahedral site hopping (out-of-plane) of TM ion. (Bottom panel) Indirect pathway employing slab gliding and hopping in the direction of out-of-plane. The moving cation’s positions are indicated by points a–g and a’–e’. (Right) The calculated energy landscape of two pathways, direct and indirect, depicted in the panels.

Recent studies reported that in-plane disordering can play a crucial role in the formation of oxygen dimers and the decay in the oxygen-redox activity for some archetypal lithium-rich electrodes^7,10,12,13,28^. Interestingly, our results for path A-1 indicate that in-plane TM disordering can be produced even in the absence of direct in-plane TM migration via sequential slab gliding and out-of-plane TM migration. The in-plane TM disordering can be formed simply by a migration of the TM ion to an adjacent vacant site in the TM layer. However, the availability of the neighboring vacant site is constrained by the amount of lithium ions that are extracted from the TM layer, which varies depending on the initial lithium-excess composition and the lithium chemical potential in the pristine structure^12^. We observed that, even with a sufficient amount of neighboring vacancies, the direct TM migration within the TM layer is not an energetically easy process, as depicted in Fig. 4a. When a TM ion moves from the initial octahedral site to the neighboring octahedral site (top panel in the figure), it must pass through either the oxygen dumbbell center or the tetrahedron between two adjacent octahedra^29^. However, the former TM migration path (path A in Fig. 4a) is quite narrow (~2.6-Å bond length of the oxygen dumbbell) and thus generally requires a high activation barrier. According to our calculation, a high activation barrier of ~2.62 eV is required for the TM to pass through the oxygen dumbbell and occupy the neighboring vacant site located in the same plane. The intermediate tetrahedron in the latter TM migration path (path B in Fig. 4a) shares at least one face with neighboring TM ions; consequently, the TM ion in the tetrahedron undergoes an exceedingly strong TM–TM repulsion, yielding an energy barrier of several eV (Fig. S10)^29^. Such high activation barriers indicate that the direct in-plane TM migrations are energetically demanding. In contrast, the in-plane disordering structure can be produced via sequential low-energy out-of-plane migrations combined with slab gliding, as illustrated in the bottom section of Fig. 4a. In this indirect pathway, the TM ion can vertically migrate between two octahedral sites (i.e., out-of-plane migration) in the migration steps of a’ to c’ or d’ to f’. Combined with the gliding processes, these migrations require only 0.99 and 0.70 eV energy barriers, respectively, as indicated in the energy profile of the figure. It is because the TM ion travels through relatively large triangle cross-sections (points b’ and e’) between two octahedra in this out-of-plane trajectory. Such a finding implies that in-plane TM migrations can occur more frequently with the series of out-of-plane TM migration events during the charge or discharge process that accompanies slab gliding such as O3-to-O1 and O1–α-to-O1–β. We also note that the ‘TM trapped in the lithium layer’ can be generated through a simpler indirect process combining slab gliding and out-of-plane TM migration in Fig. 4b. In the absence of slab gliding, the TM must make several migrating hops to form a ‘TM trapped in the lithium layer’ (top panel in Fig. 4b). To reach the final position in this case, the TM ion should pass through a triangular plane that is shared by the neighboring site, overcoming an activation barrier of approximately 1.34 eV. However, in the indirect pathway with slab gliding (bottom panel), the identical structure can be generated with only one TM hopping over the plane shared by two octahedral sites with a lower activation barrier (1.15 eV).

## Discussion

We would like to note that irreversible phase transitions generated by the combination of slab gliding and TM migration can also appear in general lithium-rich layered oxides (See Supplementary Note 1 for details). Slab gliding has been observed in a variety of stoichiometric layered oxides (i.e., ABO_2_; A: Li or Na, B: combinations of Co, Ni, and Mn)^30–33^, suggesting that it can occur in layered structures in general. However, due to the rapid structural collapses and degradation during the charge/discharge process in lithium-rich layered oxides, identifying changes in stacking sequences can be challenging. Nevertheless, it has been shown that stacking faults, which are often observed in lithium-rich layered oxides^34,35^, can promote slab gliding^17,36,37^. This is due to changes in the stacking sequence, which can reduce Coulombic energy penalty resulting from the arrangement of transition metal and lithium caused by the presence of stacking faults. Therefore, the concept of slab gliding as a cause of irreversible phase transition can be generally applicable to conventional lithium-rich layered materials considering that they can contain a significant amount of stacking faults^36^.

Slab gliding provides an easier route to form various disordered structure types by offering a greater degree of freedom to TM migration paths. It implies that the control of the slab gliding, if possible, may present a possible way to mitigate the irreversible disordering of the lithium-rich layered structure in our efforts to attain a stable oxygen-redox reaction. Although managing the sequence of slab gliding and TM migration may sound like a daunting task, we can learn from several previous studies, where potential strategies to delay or prevent the slab gliding occurrence in layered cathode materials were suggested^38–42^. For example, as widely adopted in the early studies of Li(Co, Ni)O_2_^39,43–45^, an appropriate dopant in the lithium layer can function as a pillar to inhibit the slab gliding. Elements having a similar size to lithium, such as Mg, Ca, and Cu ions, can be placed in the lithium layer, which can physically prevent the slab gliding by additional orbital hybridization with oxygen which is in the two adjoining TM layers. In fact, it has been proven that using these dopants can effectively suppress structural degradation by delaying the O1-phase formation in LiCoO_2_^39,46^. Another possible approach is to adopt the initial layered structure that allows a smaller number of stacking variations during de-/lithiations. In this regard, employing layered structures with stacking sequences other than the conventional O3-type may offer the chance to regulate the sliding sequences or timing, as previously demonstrated with O2-, P2-, or P3- type layered materials^47–50^. The transition to the O1-phase is structurally prohibited from the O2-type layered material, as it requires the major rearrangements of TM layers with the oxygen–TM bond breakings^19^. Regarding the gliding from O1–α to O1–β, it was recently found that the preferential transition to O1–β is caused by the coulombic interaction of the residual alkali ions in the alkali-metal slab with the TM slab vacancies^17^. This discovery suggests that rationally controlling the amount of excess lithium in the TM layer may weaken the driving force for the transition and mitigate the slab gliding to the O1–β stacking. However, this approach may obscure the high-energy-density merits of lithium-rich layered oxides by limiting the available capacity; therefore, the optimal amount of excess lithium should be carefully determined.

## Methods

### Computational details

Based on the DFT^51^, Vienna ab Initio simulation package (VASP) was used for the first-principles calculations. Using spin-polarized GGA + U parameterized with optB86b-vdw functional, exchange-correlation energies are evaluated to precisely match the experimentally determined interlayer distance of layered oxide. It has been demonstrated that the implemented optB86b-*vdw* exchange-correlation functional describes the experimental interlayer distance more accurately than the Perdew–Burke–Ernzerhof (PBE) functional in equivalent layered cathode materials^52,53^, and that it shows the highest performance among the various *vdw* correction functions^54^. The effective U value of 4.0 is assigned to the 4d Ru atom to address the self-interaction error^6^. We employed projector augmented wave (PAW) pseudopotentials and a plane-wave basis set with a cut-off energy of 520 eV to characterize the interaction between the ionic cores and the valence electrons. Conjugate gradient energy minimization was used to optimize all-atom position and lattice parameter until all forces on each atom are lower than 0.02 eVÅ^−1^. Brillouin zone integration was performed on a 2 × 2 × 1 *k*-point mesh for structural relaxation and on a 3 × 3 × 2 *k*-point mesh for DOS calculation.

The most basic supercell structures depending on the stacking sequence are shown in Fig. S1, and the hybrid supercell structures between O1–α and O1–β were established based on the relationship between two adjacent TMO_6_ layers. Depending on the stacking type, 30 different Li configurations were enumerated for energy calculations for the perfect layered structures at each lithium concentration. In each supercell of Li_2−*x*_RuO_3_ configured for XRD pattern generation, calculations were conducted for O3 stacking at *x* = 0 and 0.75, O1–α stacking at *x* = 1, hybrid and O1–β stacking at *x* = 1.5. All structural models were designed to have a distance of at least 10 Å between defects to rule out defect-defect interactions. Supercells with Li_0.875_RuO_3_ composition, which is shown in Fig. S5, were used to depict the combination of slab gliding and TM migration for each pathway. The most stable Li-vacancy configuration was designated among each perfect layered/disordering structure selected in the previous step to observe the change in the electronic structure of oxygen, of these, not all oxygens, but six oxygens adjacent to the Ru migration event were selected for observation.

### Material synthesis and electrode preparation

Synthesis of Li_2_RuO_3_ was performed using the conventional solid-state synthesis method. Li_2_CO_3_ (>99.0%, Sigma-Aldrich) and RuO_2_ (99.9%, Sigma-Aldrich) in stoichiometric amounts (3 g total) were used. For 24 hours, the precursors were wet ball milled in acetone. After that, the mixture underwent heat treatment at 500 °C for 5 hours and 900 °C for 12 hours while being ground in between. *N*-methyl-2-pyrrolidone (NMP; 99.5%, Sigma-Aldrich) was used to prepare the electrodes by dissolving 80 *wt*% of the active material, 10 *wt*% of polyvinylidene fluoride, and 10 *wt*% of carbon black in it. A vacuum oven set to 70 °C was used to dry the slurry for 24 hours which was cast onto aluminum foil, during which time the NMP solution evaporated. A working electrode, a lithium counter electrode, a separator (GF/F filter, Whatman), and a 1 M solution of LiPF_6_ in a solution of ethyl carbonate and dimethyl carbonate (EC/DMC, 1:1 v/v) were used to assemble cells in a glove box filled with argon.

### In situ X-ray diffraction

A coin-cell type in situ XRD cell was designed. From top to bottom, it consisted of a 125-μm-thick Be window, Al current collector layer, Li_2_RuO_3_ cathode layer, glass fiber separator, Li metal electrode, and a stainless-steel case. The Be sheet was chosen for its high X-ray transmittance and high electrochemical stability. All the components of the cell were dried in a vacuum oven at 80 °C for 12 h before cell assembly. The cell was assembled in an Ar-gas-filled glove box with H_2_O and O_2_ concentrations maintained below 1 ppm.

At room temperature, an in situ XRD investigation was done on a PANalytical X’Pert Pro diffractometer using a Cu-K_α_ source and coin-cell types cells containing Li metal anode. The current density for electrochemical cycling was 25 mA g^−1^ and the potential range was 2.0–4.6 V. Single X-ray diffraction patterns were collected for 20 minutes and continually monitored with a PIXcel 1D detector during the cycling process (PANalytical). The XRD pattern 2D images were processed with High score Plus software (PANalytical).

## Supplementary information

Supplementary Information

## Data Availability

The data supporting the findings of this study are provided in the article and its Supplementary Information and will be available from the corresponding author upon reasonable request.

## References

[1] Schmidt O, Hawkes A, Gambhir A, Staffell I (2017). The future cost of electrical energy storage based on experience rates. Nat. Energy.

[2] Cano ZP (2018). Batteries and fuel cells for emerging electric vehicle markets. Nat. Energy.

[3] Kim J-m, Jeong J-H, Jin B-S, Kim H-S (2011). Optimization of lithium in Li 1+ x [Mn 0.720 Ni 0.175 Co 0.105] O 2 as a cathode material for lithium ion battery. J. Electrochem. Sci. Technol..

[4] Seo D-H (2016). The structural and chemical origin of the oxygen redox activity in layered and cation-disordered Li-excess cathode materials. Nat. Chem..

[5] Delmas C (2016). Operating through oxygen. Nat. Chem..

[6] Sathiya M (2015). Origin of voltage decay in high-capacity layered oxide electrodes. Nat. Mater..

[7] Gent WE (2017). Coupling between oxygen redox and cation migration explains unusual electrochemistry in lithium-rich layered oxides. Nat. Commun..

[8] Assat G, Glazier SL, Delacourt C, Tarascon J-M (2019). Probing the thermal effects of voltage hysteresis in anionic redox-based lithium-rich cathodes using isothermal calorimetry. Nat. Energy.

[9] Ku K (2020). A new lithium diffusion model in layered oxides based on asymmetric but reversible transition metal migration. Energy Environ. Sci..

[10] Hong J (2019). Metal–oxygen decoordination stabilizes anion redox in Li-rich oxides. Nat. Mater..

[11] Kleiner K (2018). Origin of high capacity and poor cycling stability of Li-rich layered oxides: a long-duration in situ synchrotron powder diffraction study. Chem. Mater..

[12] House RA (2020). Superstructure control of first-cycle voltage hysteresis in oxygen-redox cathodes. Nature.

[13] House RA (2020). First-cycle voltage hysteresis in Li-rich 3 d cathodes associated with molecular O 2 trapped in the bulk. Nat. Energy.

[14] Eum D (2020). Voltage decay and redox asymmetry mitigation by reversible cation migration in lithium-rich layered oxide electrodes. Nat. Mater..

[15] Van der Ven A, Aydinol MK, Ceder G (1998). First‐principles evidence for stage ordering in Li x CoO2. J. Electrochem. Soc..

[16] de Biasi L (2019). Phase transformation behavior and stability of LiNiO2 cathode material for Li‐ion batteries obtained from in situ gas analysis and operando X‐ray diffraction. ChemSusChem.

[17] Mortemard de Boisse B (2019). Coulombic self-ordering upon charging a large-capacity layered cathode material for rechargeable batteries. Nat. Commun..

[18] Perez AJ (2016). Strong oxygen participation in the redox governing the structural and electrochemical properties of Na-rich layered oxide Na2IrO3. Chem. Mater..

[19] Radin MD, Alvarado J, Meng YS, Van der Ven A (2017). Role of crystal symmetry in the reversibility of stacking-sequence changes in layered intercalation electrodes. Nano Lett..

[20] House RA (2021). The role of O2 in O-redox cathodes for Li-ion batteries. Nat. Energy.

[21] Zheng F (2019). Impact of structural transformation on electrochemical performances of Li-rich cathode materials: the case of Li2RuO3. J. Phys. Chem. C..

[22] House RA (2021). Covalency does not suppress O2 formation in 4d and 5d Li-rich O-redox cathodes. Nat. Commun..

[23] Li B (2016). Understanding the stability for Li‐rich layered oxide Li2RuO3 cathode. Adv. Funct. Mater..

[24] Okubo M, Yamada A (2017). Molecular orbital principles of oxygen-redox battery electrodes. ACS Appl. Mater. interfaces.

[25] Song JH (2020). Anionic redox activity regulated by transition metal in lithium‐rich layered oxides. Adv. Energy Mater..

[26] Kitchaev DA, Vinckeviciute J, Van der Ven A (2021). Delocalized metal–oxygen π-redox is the origin of anomalous nonhysteretic capacity in Li-ion and Na-ion cathode materials. J. Am. Chem. Soc..

[27] Kim, B. et al. A theoretical framework for oxygen redox chemistry for sustainable batteries. *Nat. Sustain.* **5**, 708–716 (2022).

[28] Gent WE, Abate II, Yang W, Nazar LF, Chueh WC (2020). Design rules for high-valent redox in intercalation electrodes. Joule.

[29] Reed J, Ceder G (2004). Role of electronic structure in the susceptibility of metastable transition-metal oxide structures to transformation. Chem. Rev..

[30] Amatucci G, Tarascon J, Klein L (1996). CoO2, the end member of the Li x CoO2 solid solution. J. Electrochem. Soc..

[31] Croguennec L, Pouillerie C, Mansour A, Delmas C (2001). Structural characterisation of the highly deintercalated LixNi1. 02O2 phases (with x≤ 0.30) basis of a presentation given at Materials Discussion No. 3, 26–29 September, 2000, University of Cambridge, UK. J. Mater. Chem..

[32] Yin S-C, Rho Y-H, Swainson I, Nazar L (2006). X-ray/neutron diffraction and electrochemical studies of lithium de/re-intercalation in Li1-x Co1/3Ni1/3Mn1/3O2 (x= 0→ 1). Chem. Mater..

[33] Wang PF, You Y, Yin YX, Guo YG (2018). Layered oxide cathodes for sodium‐ion batteries: phase transition, air stability, and performance. Adv. Energy Mater..

[34] Li Q (2019). Dynamic imaging of crystalline defects in lithium-manganese oxide electrodes during electrochemical activation to high voltage. Nat. Commun..

[35] Boulineau A, Croguennec L, Delmas C, Weill F (2009). Reinvestigation of Li2MnO3 structure: electron diffraction and high resolution TEM. Chem. Mater..

[36] Singer A (2018). Nucleation of dislocations and their dynamics in layered oxide cathode materials during battery charging. Nat. Energy.

[37] Wang R (2013). Atomic structure of Li2MnO3 after partial delithiation and re‐lithiation. Adv. Energy Mater..

[38] Pouillerie VC, Croguennec L, Delmas C (2000). The LixNi1− yMgyO2 (y= 0.05, 0.10) system: structural modifications observed upon cycling. Solid State Ion..

[39] Huang Y (2021). Mg‐pillared LiCoO2: towards stable cycling at 4.6 V. Angew. Chem..

[40] Chu S (2021). Pinning effect enhanced structural stability toward a zero‐strain layered cathode for sodium‐ion batteries. Angew. Chem..

[41] Wang C (2021). Tuning local chemistry of P2 layered-oxide cathode for high energy and long cycles of sodium-ion battery. Nat. Commun..

[42] Xie Y (2016). In operando XRD and TXM study on the metastable structure change of NaNi1/3Fe1/3Mn1/3O2 under electrochemical sodium‐ion intercalation. Adv. Energy Mater..

[43] Yu Y (2021). Lattice Modulation by Ca/P dual-doping for fast and stable Li+ intercalation/extraction in high-voltage LiCoO2. J. Phys. Chem. C..

[44] Qin, N. et al. Hierarchical doping engineering with active/inert dual elements stabilizes LiCoO2 to 4.6 V. *Adv. Energy Mater.* **12**, 2201549.

[45] Tarascon J (1999). In situ structural and electrochemical study of Ni1− xCoxO2 metastable oxides prepared by soft chemistry. J. Solid State Chem..

[46] Zhang J-N (2019). Trace doping of multiple elements enables stable battery cycling of LiCoO2 at 4.6 V. Nat. Energy.

[47] Paulsen JM, Mueller-Neuhaus JR, Dahn JR (2000). Layered LiCoO[sub 2] with a different oxygen stacking (O2 structure) as a cathode material for rechargeable lithium batteries. J. Electrochem. Soc..

[48] Somerville JW (2019). Nature of the “Z”-phase in layered Na-ion battery cathodes. Energy Environ. Sci..

[49] Sun Y, Guo S, Zhou H (2019). Adverse effects of interlayer-gliding in layered transition-metal oxides on electrochemical sodium-ion storage. Energy Environ. Sci..

[50] Gao A (2022). Topologically protected oxygen redox in a layered manganese oxide cathode for sustainable batteries. Nat. Sustain..

[51] Kresse G, Furthmüller J (1996). Efficient iterative schemes for ab initio total-energy calculations using a plane-wave basis set. Phys. Rev. B..

[52] Aykol M, Kim S, Wolverton C (2015). Van der Waals interactions in layered lithium cobalt oxides. J. Phys. Chem. C..

[53] Yoshida T, Hongo K, Maezono R (2019). First-principles study of structural transitions in LiNiO2 and high-throughput screening for long life battery. J. Phys. Chem. C..

[54] Klimeš J, Bowler DR, Michaelides A (2011). Van der Waals density functionals applied to solids. Phys. Rev. B..

