# Supplementary material for: Slab gliding, a hidden factor that induces irreversibility and redox asymmetry of lithium-rich layered oxide cathodes
Source: Nat Commun. 2023 Jul 12;14:4149. doi: 10.1038/s41467-023-39838-y (PMC10338458; doi:10.1038/s41467-023-39838-y)
Supplement: Supplementary file 1 — Supplementary Information [file 41467_2023_39838_MOESM1_ESM.pdf]

Supplementary Information

Slab gliding, a hidden factor that induces irreversibility and redox asymmetry of lithium-rich layered oxide cathodes

*Jun-Hyuk Song[1,‡,†], Seungju Yu[1,†], Byunghoon Kim[1], Donggun Eum[1], Jiung Cho[2], Ho-Young Jang[1], Sung-O Park[1], Jaekyun Yoo[1], Youngmin Ko[1], Kyeongsu Lee[1], Myeong Hwan Lee[1], Byungwook Kang[1], and Kisuk Kang [1,3,4,5] **

[1]Department of Materials Science and Engineering, Research Institute of Advanced Materials (RIAM), Seoul National University, 1 Gwanak-ro, Gwanak-gu, Seoul 151-742, Republic of Korea.

[2]Western Seoul Center, Korea Basic Science Institute, 150 Bugahyeon-ro, Seoul 03759 Republic of Korea

[3]Center for Nanoparticle Research, Institute for Basic Science (IBS), Seoul National University, 1 Gwanak-ro, Gwanak-gu, Seoul 151-742, Republic of Korea.

[4]Institute of Engineering Research, College of Engineering, Seoul National University, 1 Gwanak-ro, Gwanak-gu, Seoul 151-742, Republic of Korea

[5]School of Chemical and Biological Engineering, and Institute of Chemical Process, Seoul National University, Seoul 08826, Republic of Korea

[‡]Present address: LiB Materials Research Group, Research Institute of Industrial Science & Technology (RIST), 100 Songdogwahak-ro, Yeonsu-gu, Incheon, Republic of Korea

[†] These authors contributed equally to this paper

* Corresponding author. E-mail: matlgen1@snu.ac.kr

Supplementary Note 1: Validating generality of our slab gliding theory

Even though there is little experimental evidence that lithium-rich electrodes undergo a glide of their transition metal layers, stacking faults are frequently observed in these lithium-rich layered oxide cathodes[1,2]. Moreover, stacking faults in certain materials can change in size during electrochemical cycling, as established by studies[3-5] and so on. Singer *et al.* directly observed the nucleation of a mobile dislocation network[3] (*e.g.*, the mixture of O3 and O1 stackings with extra half-plane) in lithium-rich layered oxides ($Li_{1.2}Ni_{0.133}Mn_{0.533}Co_{0.133}O_2$) using operando three-dimensional Bragg coherent diffractive imaging. They also calculated the dislocation density, which increases up to 10^{10} cm^{-2} in $Li_{1.2}Ni_{0.133}Mn_{0.533}Co_{0.133}O_2$ when charged to 4.4 V (*vs*. Li/Li^+). It was also stated that "*At such a high value, we anticipate a sizable impact on the material's performance, particularly on voltage fade and oxygen activity*." While these studies do not further discuss the effect of the mixture of O3 and O1 stackings, the substantial increase of the dislocation density upon charging indicates that the stacking faults are generated during the charge and discharge, which will inevitably involve the slab gliding process. Furthermore, in more recent report[5], it was shown that stacking faults trigger the slab gliding process to minimize Coulombic energy, and a self-repairing of stacking faults results in much better reversibility of the Na_2RuO_3 electrode reaction. These series of reports suggest that the slab gliding process is not only feasible in our model Li_2RuO_3, but it also appears to be a common occurrence in lithium-rich layered materials, which requires further investigation to understand its correlation with the electrochemical reversibility of such materials.

In order to further justify the generality of the slab gliding process in conventional lithium-rich layered oxide, we considered a nickel/manganese-based lithium rich layered oxide, $Li_{1.17-}$

$_{x}Mn_{0.58}Ni_{0.25}O_2$, possessing O1-like stacking faults. Fig. S11 shows how the migration of transition metals is affected by the presence of the O1 stacking in the conventional O3 structure. Four different scenarios were examined for $Li_{1.17-x}Mn_{0.58}Ni_{0.25}O_2$ similar to the case of Li_2RuO_3 in the main text. It reveals that, in accordance with the results of Li_2RuO_3, the slab gliding considerably promotes the transition metal migration in the layered structure. Moreover, irreversible pathways (**Paths 3** and **4**) become far more stable through slab gliding than reversible ones, resulting in disordered O3-type structures containing the displacement of transition metal. These results are consistent with the previous research which reported the observation of transition metal trapped in lithium layer[6] (**Path 3**) and in-plane disordering[7] (**Path 4**) after electrochemical cycling, and suggest that the transition metal migration aided by the slab gliding is applicable not only to Li_2RuO_3 but also to nickel/manganese-based lithium rich layered oxide.

The results suggest that structural degradation can be intensified by the combination of transition metal migration and slab gliding caused by stacking faults in lithium-rich layered oxide cathodes. Revealing the effect of gliding highlights that it is critical to regulate/suppress gliding transformations, which has not been previously considered, in order to preserve the initial structure and eliminate the irreversibility and redox asymmetry of lithium-rich layered oxide cathodes. We would like to emphasize that this can be applied not only to a specific material but also to a wide range of lithium-rich layered oxide cathodes.

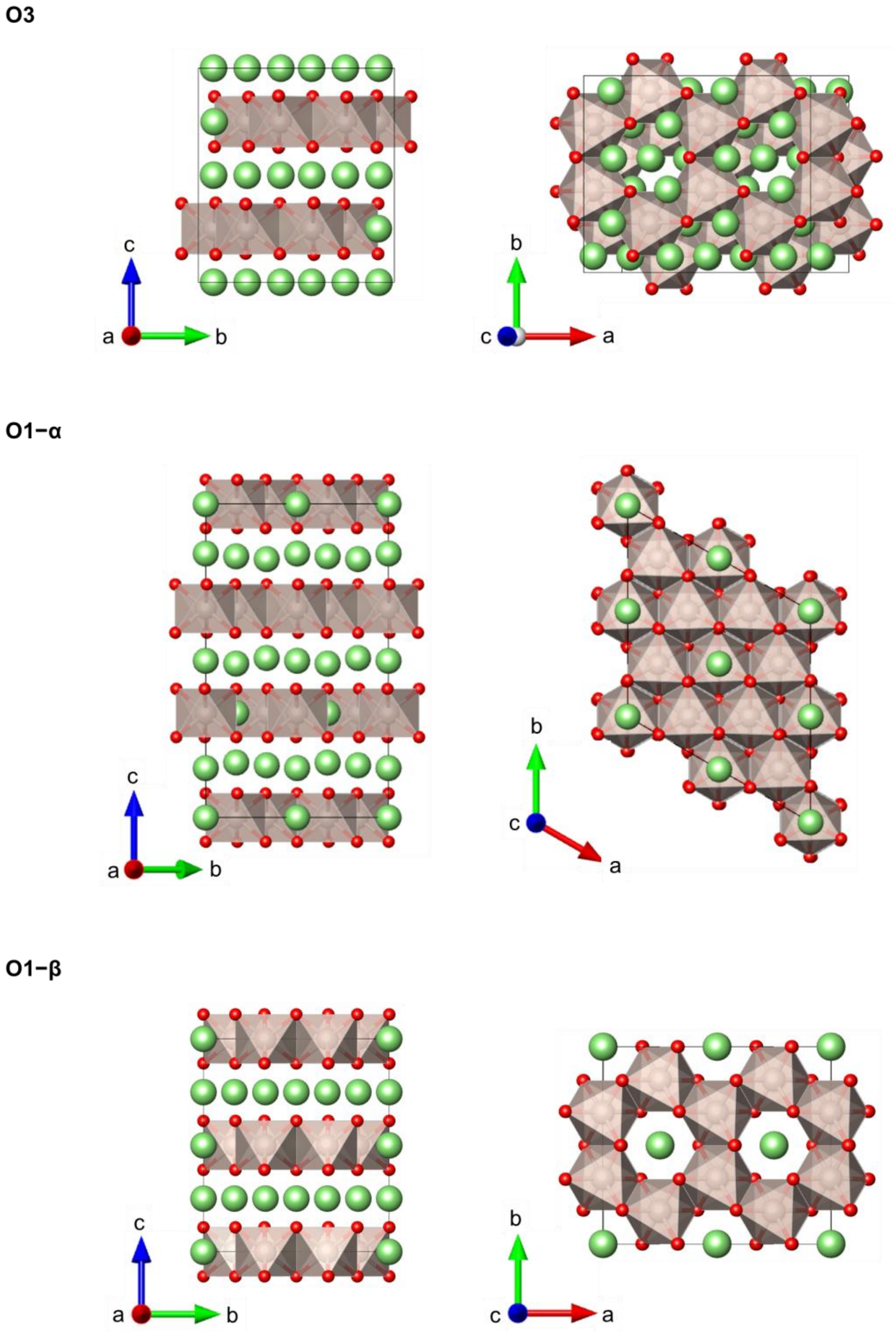

Figure S1. Supercell structures of $Li_{2-x}RuO_3$ (x = 0) with three different stacking sequences.

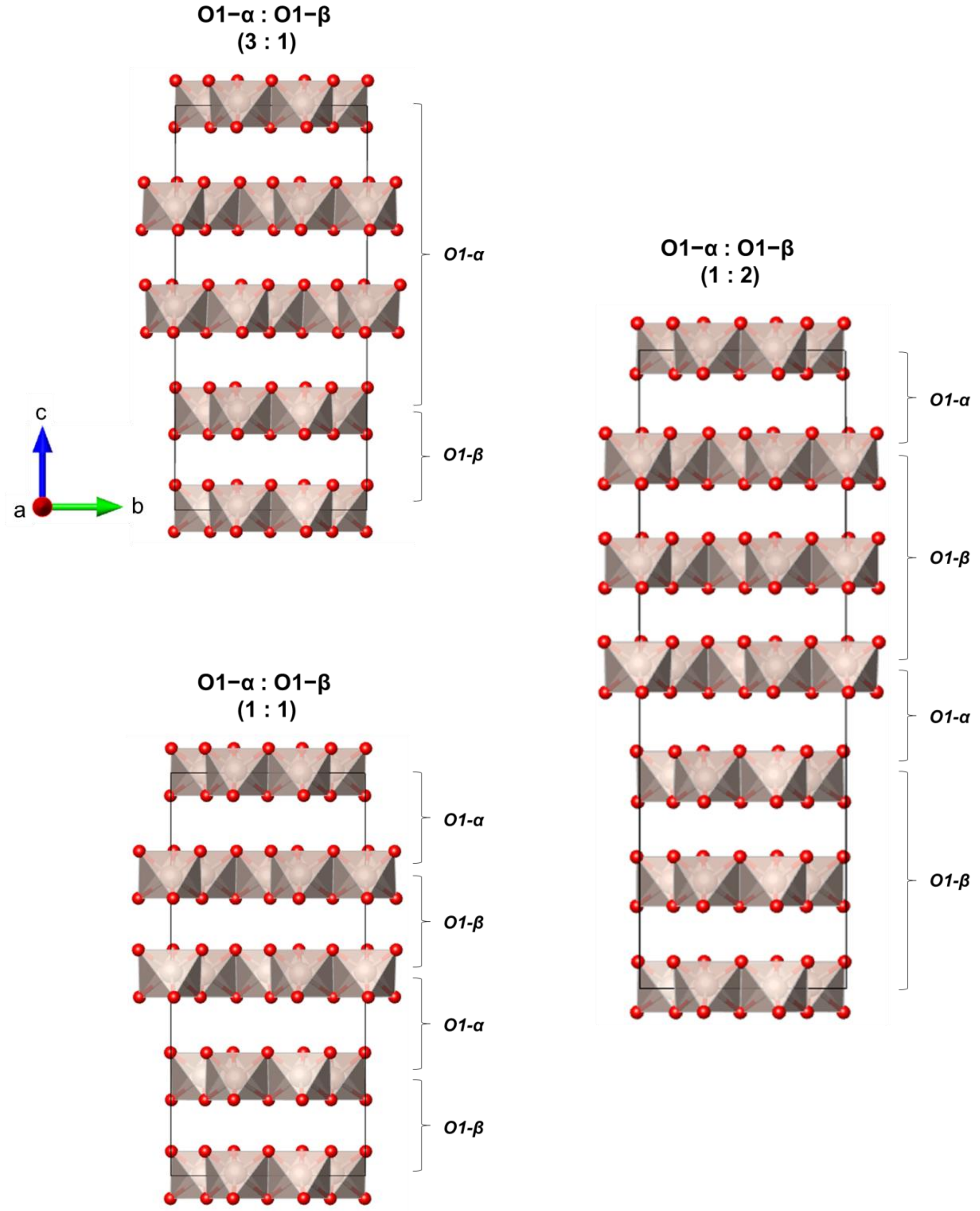

Figure S2. Hybrid supercell structures of three different $Li_{2-x}RuO_3$ with two kinds of stacking (O1–α, O1–β) motif. Lithium ions are omitted.

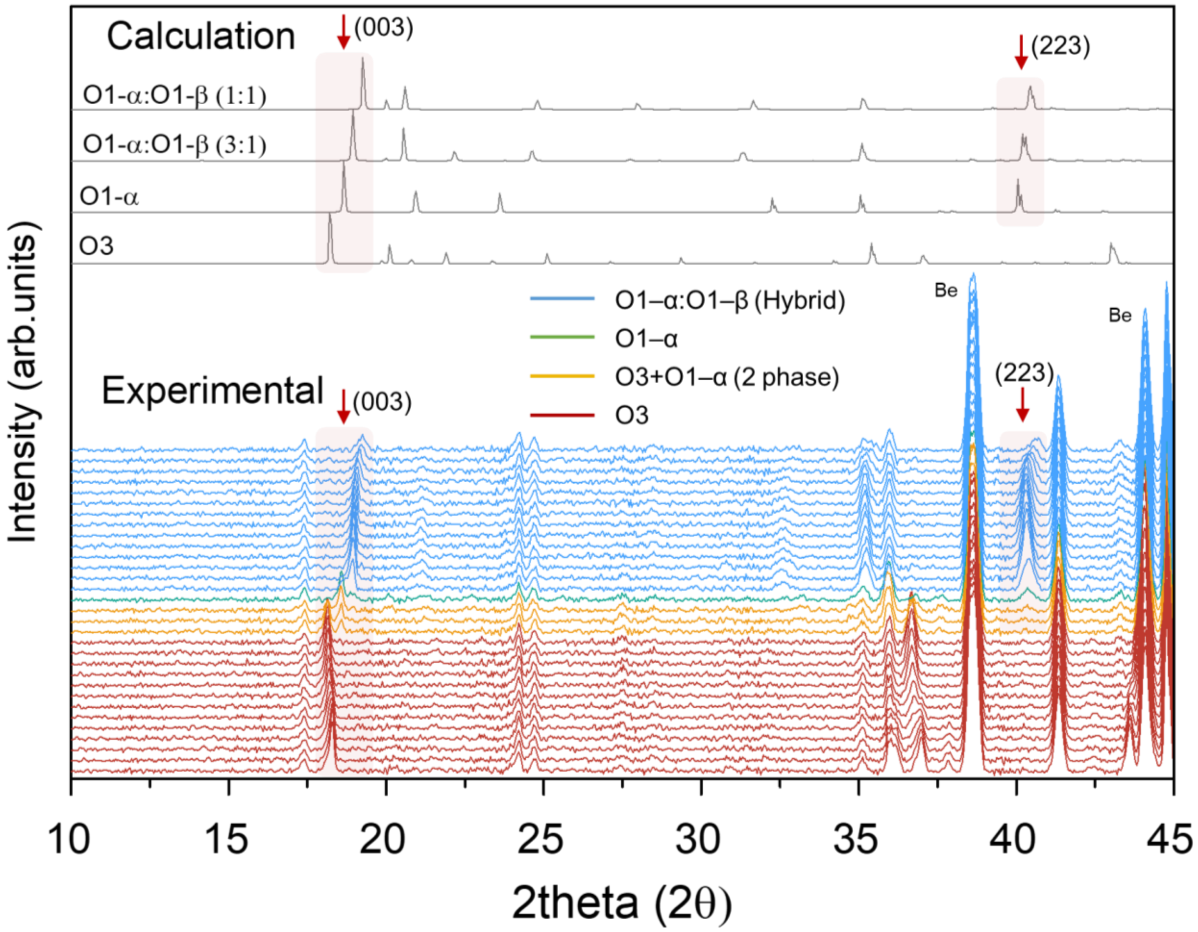

Figure S3. Comparison of calculated X-ray diffraction pattern and operando X-ray diffraction pattern for the $Li_{2-x}RuO_3$ electrode during the first charge.

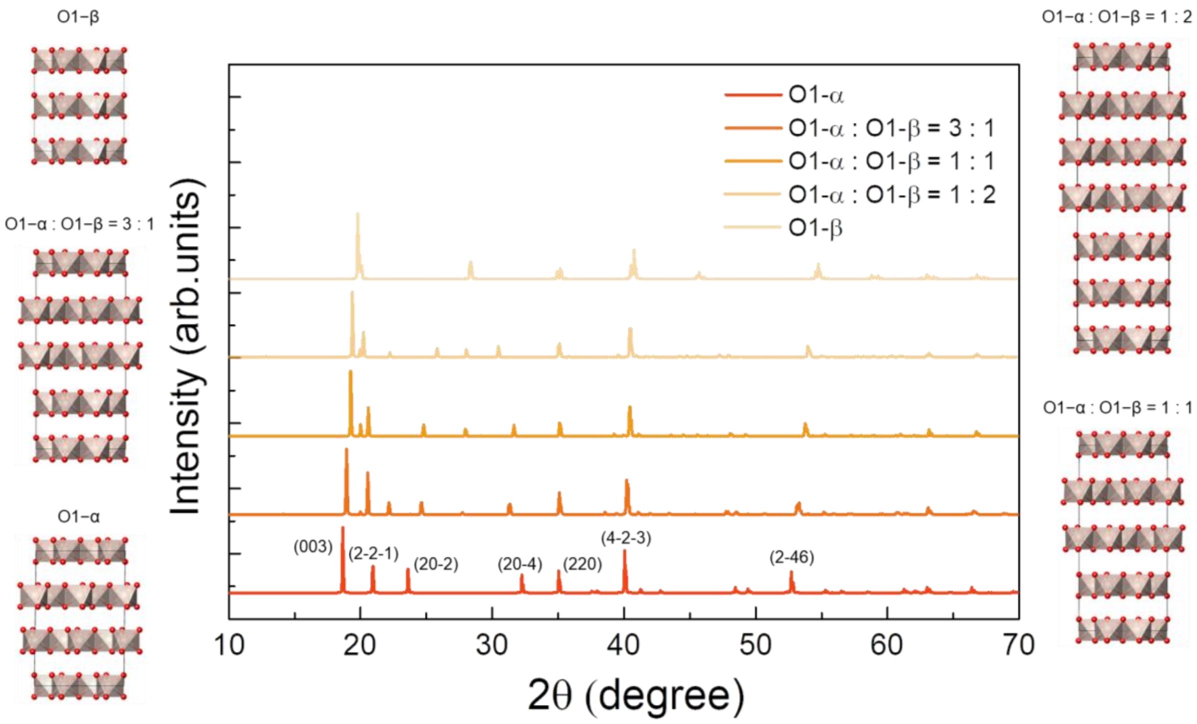

Figure S4. Calculated X-ray diffraction pattern of hybrid $Li_{2-x}RuO_3$ electrodes with different O1–α and O1–β contents.

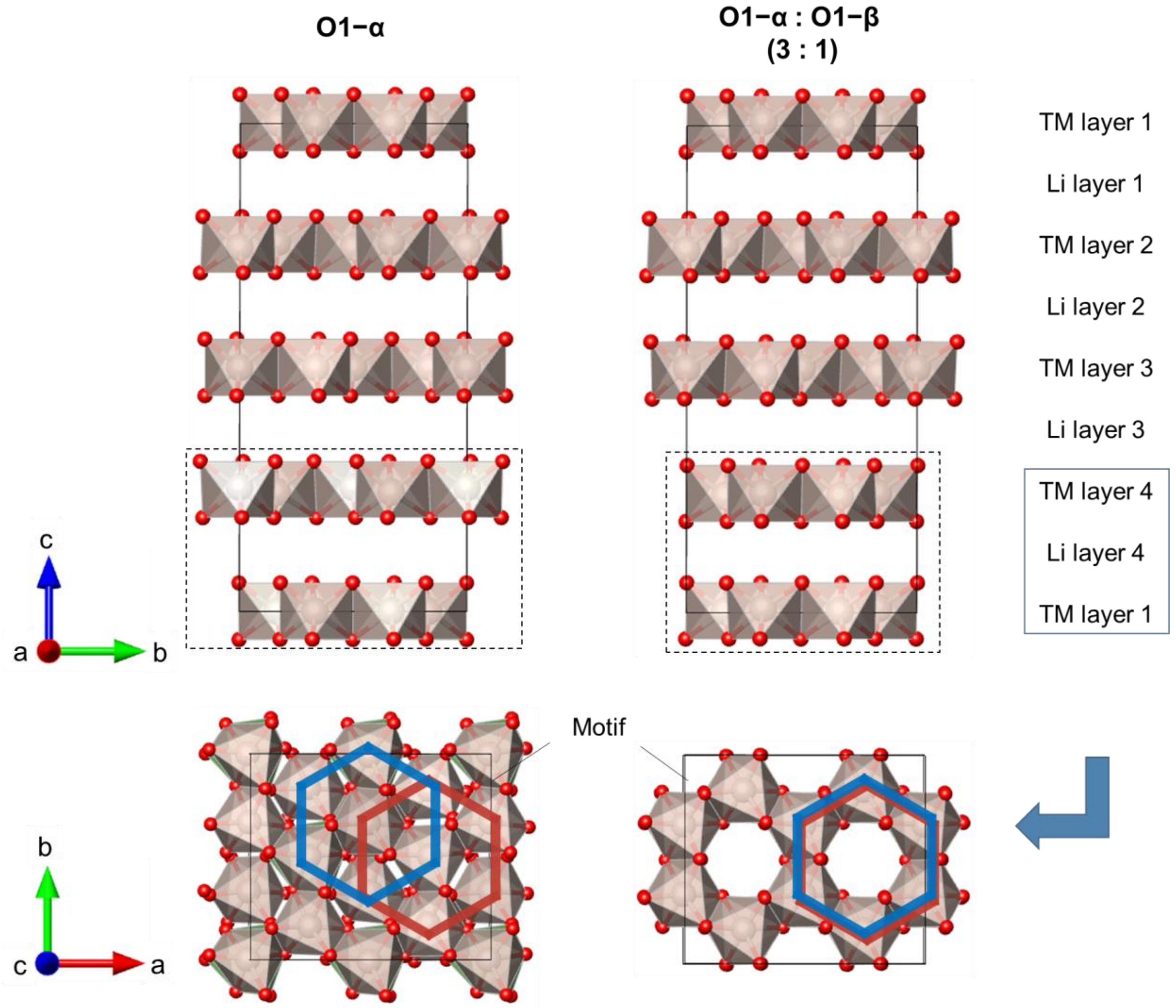

Figure S5. Two different O1−α stacking and O1−α : O1−β = 3 : 1 hybrid stacking in $Li_{2-x}RuO_3$ (x = 1.25) prepared for calculation. Lithium ions are omitted.

Charge process

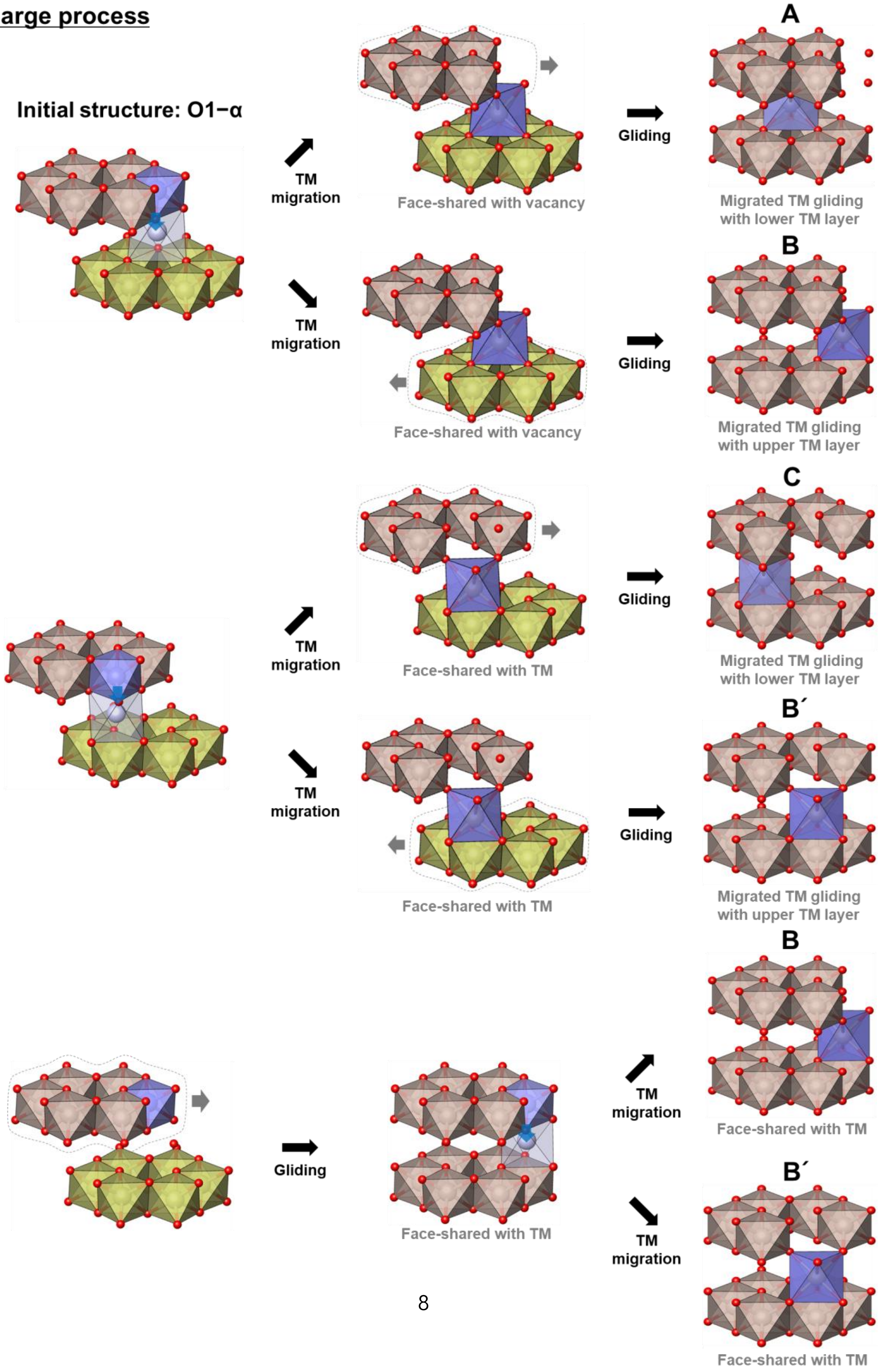

Discharge process

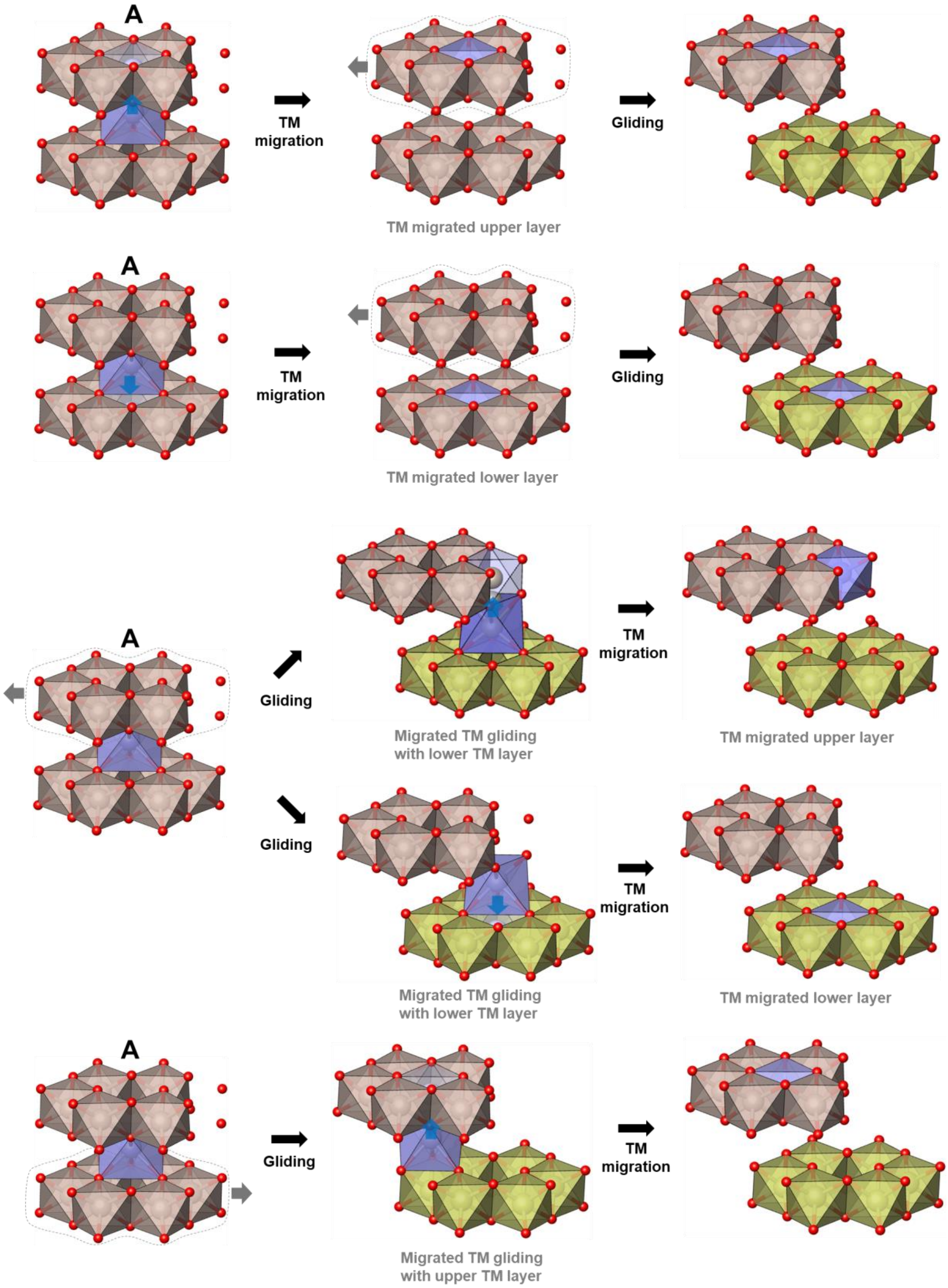

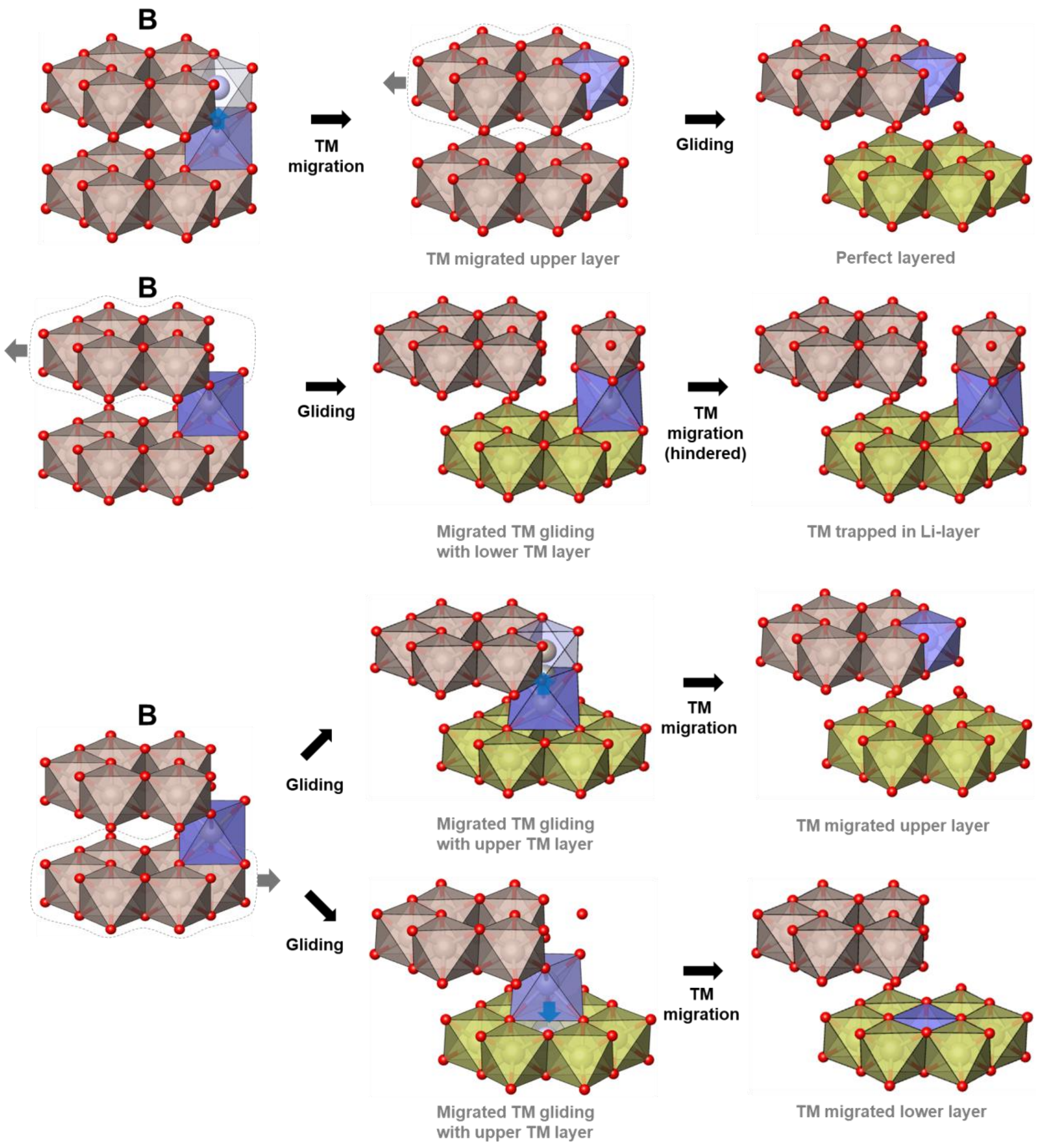
B
TM
migration
TM migrated upper layer
Gliding
Perfect layered
B
Gliding
Migrated TM gliding
with lower TM layer
TM
migration
(hindered)
TM trapped in Li-layer
B
Gliding
Migrated TM gliding
with upper TM layer
TM
migration
TM migrated upper layer
Gliding
Migrated TM gliding
with upper TM layer
TM
migration
TM migrated lower layer

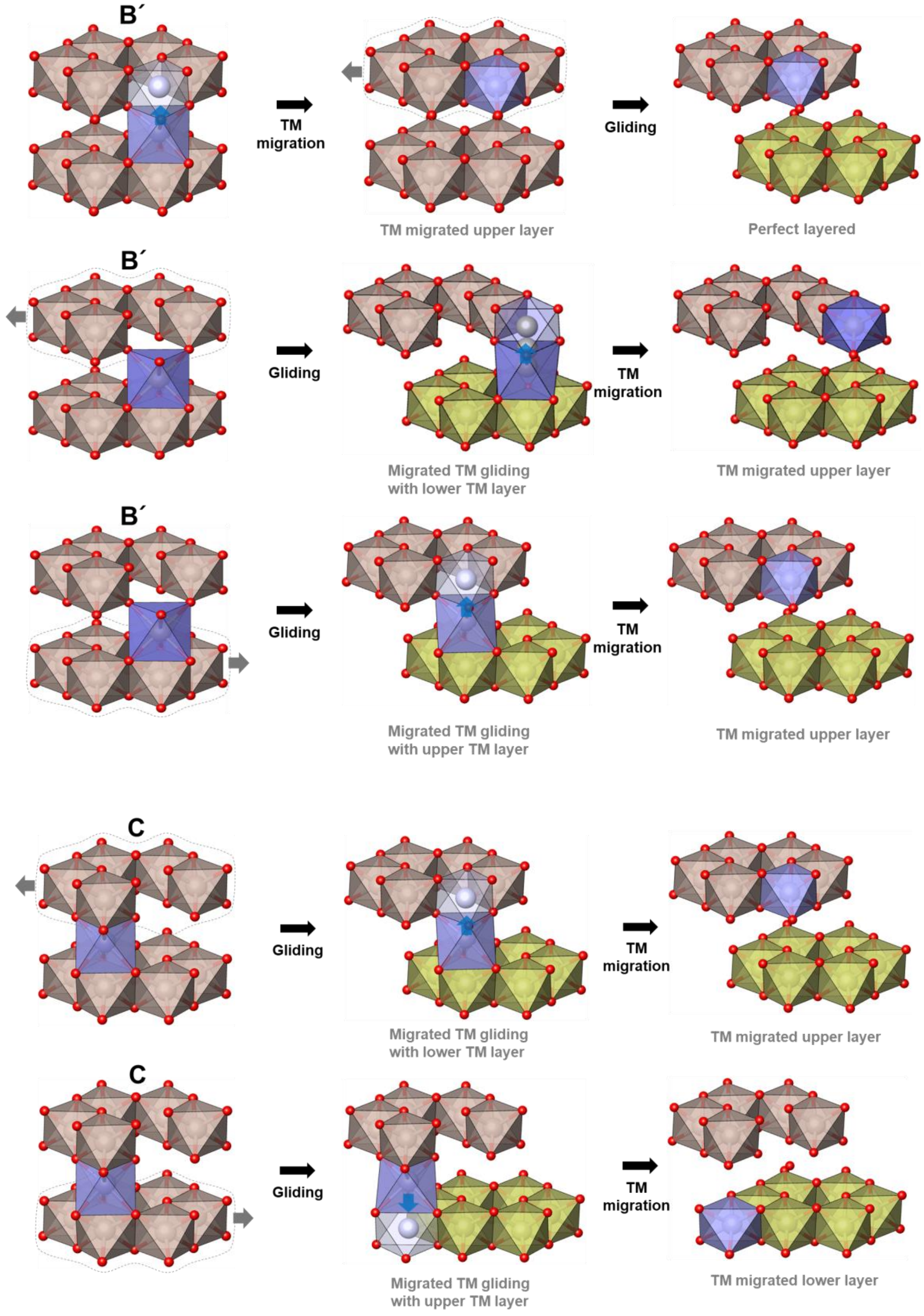
B´
TM migration
Gliding
TM migrated upper layer
Perfect layered
B´
Gliding
TM migration
Migrated TM gliding with lower TM layer
TM migrated upper layer
B´
Gliding
TM migration
Migrated TM gliding with upper TM layer
TM migrated upper layer
C
Gliding
TM migration
Migrated TM gliding with lower TM layer
TM migrated upper layer
C
Gliding
TM migration
Migrated TM gliding with upper TM layer
TM migrated lower layer

Figure S6. Structural evolution map during charging and discharging. The total path includes 6 structural evolution paths during the charging process, and 14 structural evolution paths during the discharging process.

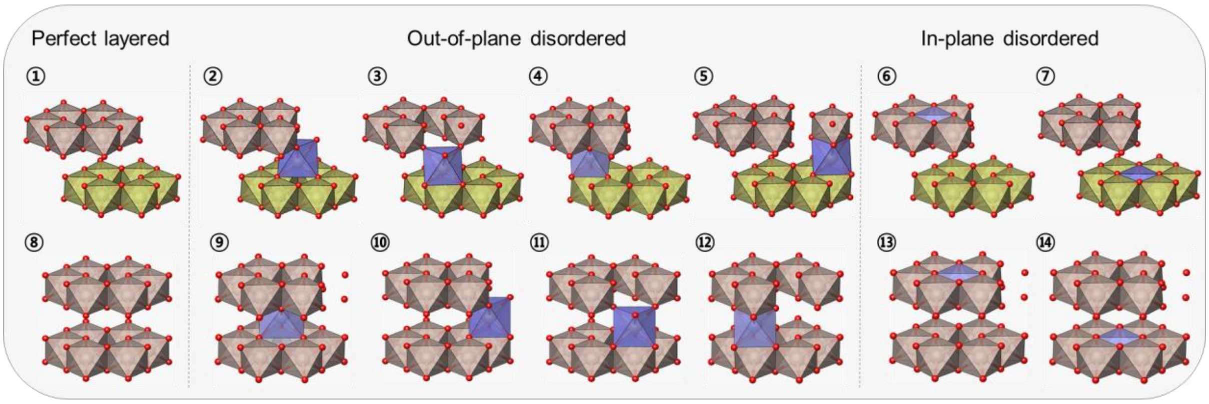

Figure S7. All kinds of disordered structures that can be formed by slab gliding and out-of-plane TM migration during charging and discharging.

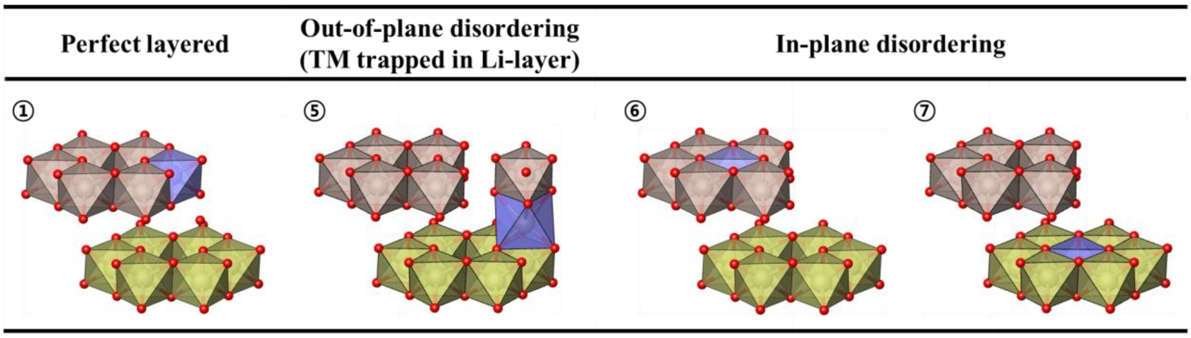

Figure S8. Structures that can be formed through a total of 20 pathways after charging and discharging process.

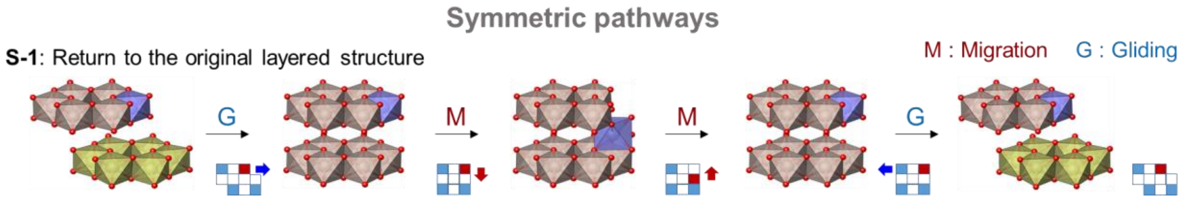

Figure S9. Representative symmetric structural evolutionary pathways

	Symmetric path	
	Charge	Discharge
Path 1	① → ② → ⑨	⑨ → ② → ①
Path 2	① → ② → ⑩	⑩ → ② → ①
Path 3	① → ③ → ⑪	⑪ → ③ → ①
Path 4	① → ③ → ⑫	⑫ → ③ → ①
Path 5	① → ⑧ → ⑩	⑩ → ⑧ → ①

	Asymmetric path	
	Charge	Discharge
Path 6	① → ② → ⑨	⑨ → ⑬ → ⑥
Path 7	① → ② → ⑨	⑨ → ⑭ → ⑦
Path 8	① → ② → ⑨	⑨ → ② → ⑦
Path 9	① → ② → ⑨	⑨ → ④ → ⑥
Path 10	① → ② → ⑩	⑩ → ⑧ → ①
Path 11	① → ② → ⑩	⑩ → ⑤ → ⑤
Path 12	① → ② → ⑩	⑩ → ② → ⑦
Path 13	① → ③ → ⑪	⑪ → ⑧ → ①
Path 14	① → ③ → ⑪	⑪ → ④ → ⑥
Path 15	① → ③ → ⑫	⑫ → ④ → ⑦
Path 16	① → ⑧ → ⑩	⑩ → ② → ①
Path 17	① → ⑧ → ⑩	⑩ → ⑤ → ⑤
Path 18	① → ⑧ → ⑩	⑩ → ② → ⑦
Path 19	① → ⑧ → ⑪	⑪ → ③ → ①
Path 20	① → ⑧ → ⑪	⑪ → ④ → ⑥

Table S1. All paths that can be formed by slab gliding and out-of-plane TM migration during charging and discharging. The total path includes 5 symmetric structural evolution paths and 15 asymmetric structural evolution paths.

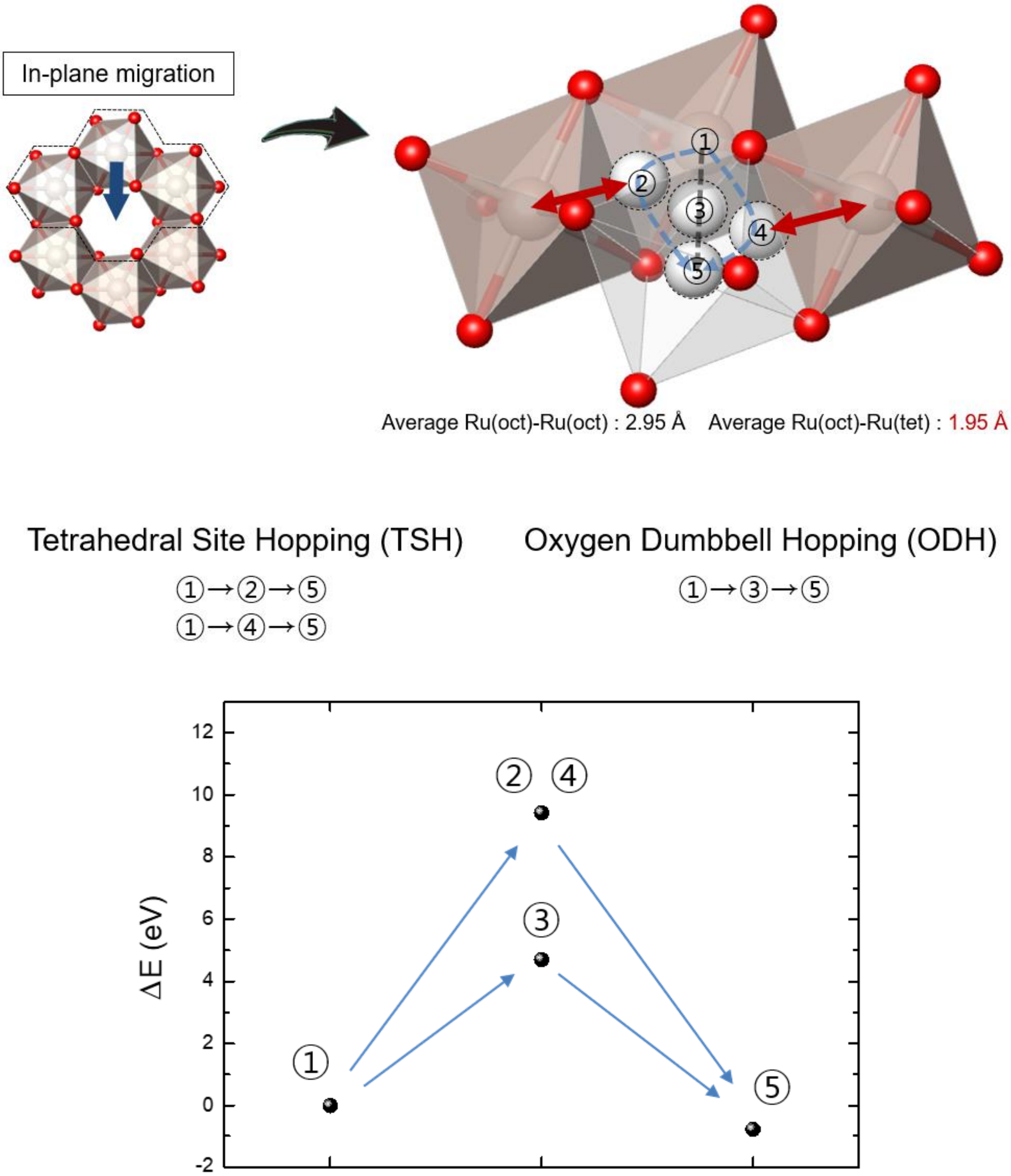

Figure S10. Schematics of two plausible pathways (TSH and ODH) of direct in-plane TM migration and the energy of these pathways in fixed atom position.

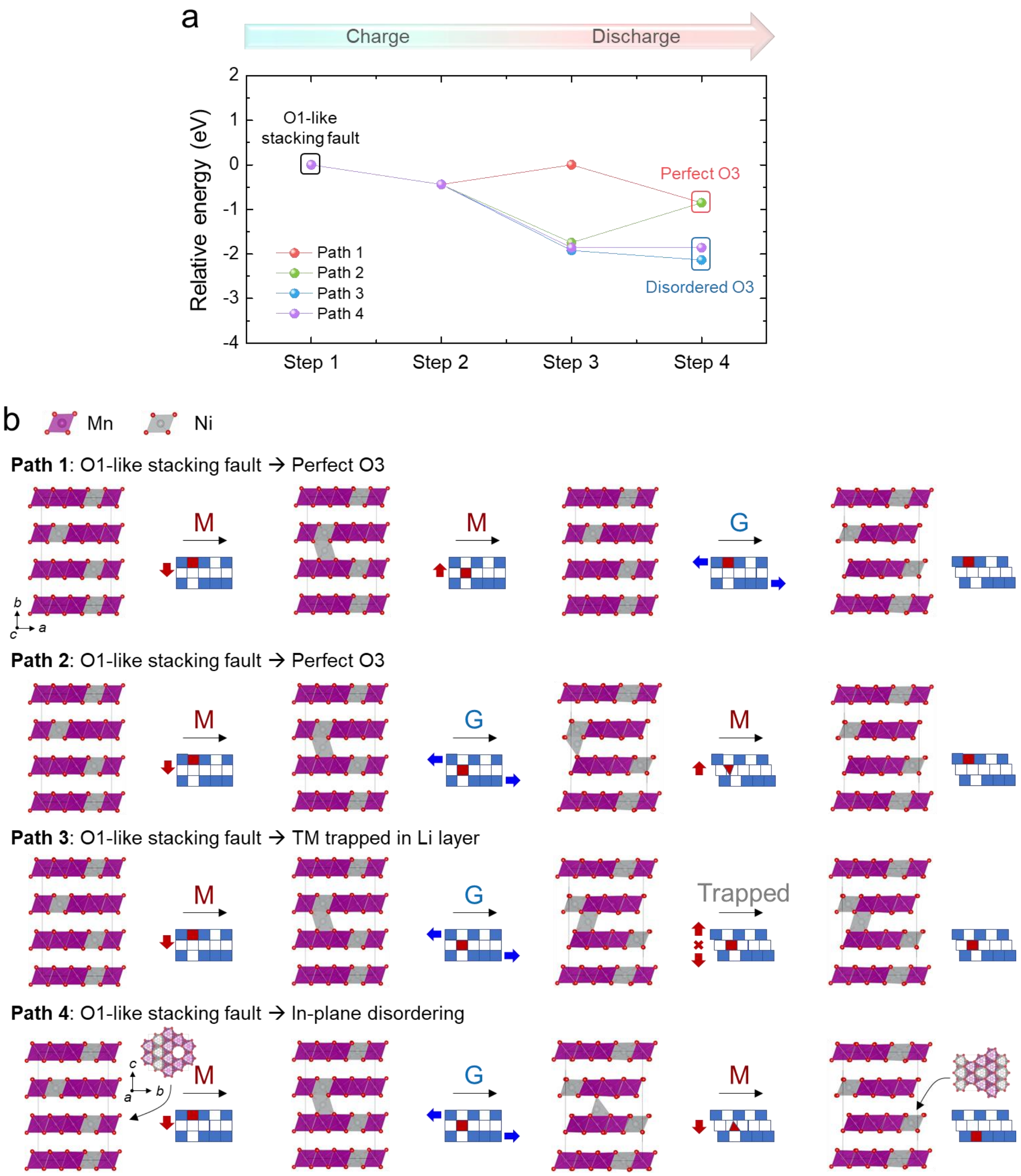

Figure S11. Validation of the effect of slab gliding on irreversible phase transition of other lithium-rich layered oxide cathodes ($Li_{1.17-x}Mn_{0.58}Ni_{0.25}O_2$). a Relative energy of each path

(reversible pathway for **Paths 1** and **2** and irreversible pathway for **Paths 3** and **4**). **b** Structural illustration of each pathway.

References

1 Boulineau, A., Croguennec, L., Delmas, C. & Weill, F. Reinvestigation of Li2MnO3 structure: electron diffraction and high resolution TEM. *Chemistry of Materials* **21**, 4216-4222 (2009).

2 Li, Q. *et al.* Dynamic imaging of crystalline defects in lithium-manganese oxide electrodes during electrochemical activation to high voltage. *Nature communications* **10**, 1692 (2019).

3 Singer, A. *et al.* Nucleation of dislocations and their dynamics in layered oxide cathode materials during battery charging. *Nature Energy* **3**, 641-647 (2018).

4 Wang, R. *et al.* Atomic structure of Li2MnO3 after partial delithiation and re-lithiation. *Advanced Energy Materials* **3**, 1358-1367 (2013).

5 Mortemard de Boisse, B. *et al.* Coulombic self-ordering upon charging a large-capacity layered cathode material for rechargeable batteries. *Nature Communications* **10**, 2185 (2019).

6 Sathiya, M. *et al.* Origin of voltage decay in high-capacity layered oxide electrodes. *Nature materials* **14**, 230-238 (2015).

7 House, R. A. *et al.* Superstructure control of first-cycle voltage hysteresis in oxygen-redox cathodes. *Nature* **577**, 502-508 (2020).
